# Odds of talking to healthcare providers as the initial source of healthcare information: updated cross-sectional results from the Health Information National Trends Survey (HINTS)

**DOI:** 10.1186/s12875-018-0805-7

**Published:** 2018-08-29

**Authors:** Christine M. Swoboda, Joseph M. Van Hulle, Ann Scheck McAlearney, Timothy R. Huerta

**Affiliations:** 1 Department of Family Medicine, The Ohio State University, Room 502, 460 Medical Center Drive, Columbus, OH 43210 USA; 2 Department of Family Medicine, The Ohio State University, Room 530, 460 Medical Center Drive, Columbus, OH 43210 USA; 3 Departments of Family Medicine and Biomedical Informatics, The Ohio State University, Room 532, 460 Medical Center Drive, Columbus, OH 43210 USA

**Keywords:** Health information sources, Information seeking behavior, Health communication, Healthcare providers, Cross-sectional

## Abstract

**Background:**

People use a variety of means to find health information, including searching the Internet, seeking print sources, and talking to healthcare providers, family members, and friends. Doctors are considered the most trusted source of health information, but people may be underutilizing them in favor of searching the Internet.

**Methods:**

A multinomial logistic regression of cross-sectional data from Cycle 4 of the Health Information National Trends Survey (HINTS) was conducted. Independent variables included gender, age, rurality, cancer history, general health, income, race, education level, insurance status, veteran status, Internet use, and data year; the dependent variable was the first chosen source of health information.

**Results:**

The most frequent initial source of health information was the Internet, and the second most frequent was healthcare providers. There were significant differences in odds of using healthcare providers as the first source of health information. Those likely to use doctors as their initial source of health information were older adults, black adults, adults with health insurance, those who do not use the Internet, and adults who do not have a college degree.

**Conclusions:**

People who use healthcare providers as the first source of health information may have better access to health care and be those less likely to use the Internet. Doctors may have to provide more information to those who do not use the internet and spend time verifying information for those who do use health information from the internet.

## Background

Individuals want information to manage and improve their health. While the most trusted source of health information is healthcare providers [1], individuals draw on a number of resources for health information. Prior research has found that the Internet is the fastest growing source of health information [2], and is used more among younger adults, people with chronic conditions, the uninsured, and people who live far from their doctor [3, 4].

Despite its widespread use, individuals also frequently distrust information they find on the Internet, seeking to verify the information obtained with a doctor, through other websites, print sources, or with friends and family [5]. This distrust is well warranted - researchers have shown that the quality of information provided on the Internet varies widely by website, and that there is potential for harm due to inaccurate information [6]. As a result, individuals rely on a diversity of resources to help address their health information needs.

Physicians have a vested interest in being a primary and trusted source of health information. Discussing health information strengthens the patient-provider relationship; research indicates that patients appreciate when physicians help them evaluate health information they received from other sources [7]. Further, research has confirmed the importance of timely access to health information to allow patients to make informed treatment decisions at their medical appointments [8] and treatment satisfaction helps encourage adherence and positive health outcomes [9]. Patients prefer to be involved in their own medical decision-making, and the more informed they feel, the more involved they prefer to be in decision-making [10] and the more satisfied they feel with treatment decisions [11, 12]. Further, healthcare providers are often in the only position to know whether the information that individuals receive are appropriate for their conditions, ensuring patients are informed and are using accurate information when making decisions.

Given the diversity of sources of information and its widely varied quality, it is critical that we keep up on trends associated with changes in how individuals source their health information. This analysis updates prior work conducted by Volkman, Luger, Harvey, Hogan, Shimada, Amante, McInnes, Feng, and Houston in 2014 concerning the most frequently used first sources of health information, and characteristics of patients that seek health information from doctors first [13]. The demographic trends among those who seek doctors as the first source of health information are analyzed using expanded data to identify substantive changes in practice.

## Methods

### Data collection

This study used data obtained through the Health Information National Trends Survey (HINTS 4) [14], an annual survey fielded to a representative sample of United States (U.S.) adults over 18 years of age, sponsored by the National Cancer Institute (NCI), which explores the public’s use of cancer-related information. The fourth version of HINTS, used in this study, was administered by mail to a sample of U.S. civilian, non-institutionalized adults in four separate cycles: 2011, 2012, 2013, and 2014. HINTS had a response rate of 37% in HINTS 4 Cycle 1, 40% in Cycle 2, 35% in Cycle 3, and 34% in Cycle 4. [15, 16] Non-response was systematically more likely for respondents who were male, minority, younger, less educated, or Hispanic. The survey uses a stratified postal address frame to randomly sample residential addresses. Weighting is provided to allow interested researchers to develop population estimates.

### Participants

A population sample was obtained from the combined 2011–2014 HINTS data. Of the total 14,451 participants, 10,024 were included in this study. The exclusion criteria, mirrored from *Volkman* et al. *2014,* allow for comparison and extension on prior research and were based on responses to the survey question: “The most recent time you looked for information about health or medical topics, where did you go first?” Participants were excluded if they had a missing response (*n* = 112), a response error (e.g., multiple responses) (*n* = 1848), or if they never sought health or medical topic information (i.e., question inapplicable) (*n* = 2467).

### Measures

To quantify the *first source* used for seeking health information, participants were asked: “The most recent time you looked for information about health or medical topics, where did you go first?” Potential responses to this question included: Internet, doctor/healthcare provider, publications, family/friends/co-workers, telephone service, cancer organization, library, complementary, alternative, or unconventional medicine practitioner, and other. A derived variable was created by dichotomizing the responses as “doctor/healthcare provider” and “other” (i.e., Internet, publications, family/friends/co-workers, other, telephone service, cancer organization, library, and complementary or alternative medicine practitioner).

Sociodemographic characteristics used in this analysis included *gender* (male/female), *age* (18–45/ 46–64/65–75/ 76≤), *income* (<$20,000 / $20,000–$49,999 / $50,000≤), *race/ethnicity* (non-Hispanic White/non-Hispanic Black/Hispanic/Other), *education* (<high school/high school graduate/some college/college graduate≤), *rurality*, and *veteran status*. *Rurality* (yes/no) was determined by the 2003 USDA rural/urban designation assigned to the respondent’s mailing address [17]. *Veteran status* (yes/no) was determined by responses to the following question: “Have you ever served on active duty in the U.S. Armed Forces, military Reserves, or National Guard? Active duty does not include training in the Reserves, or National Guard, but DOES include activation, for example, for the Persian Gulf War.”

*Cancer history*, *general health*, and *health insurance* were included in the analysis as important clinical characteristics. *Cancer history* (yes/no) was assessed by the question: “Have you ever been diagnosed with cancer?” The question: “In general, would you say your health is? [excellent/very good/good/fair/poor]” was used to assess *general health* (excellent/very good/good/fair/poor). In 2011 and 2013 participants were categorized as possessing insurance if they had: “Insurance through a current or former employer or union,” “Insurance purchased directly from an insurance company,” “Medicare, Medicaid, Medical Assistance, or any kind of government-assistance plan for those with low incomes or a disability,” “TRICARE or other military health care,” “VA health care,” or “Indian Health Service.” In survey years 2012 and 2014 possession of health insurance was determined by the question: “Do you have any kind of health care coverage, including health insurance, prepaid plans such as HMOs, or government plans such as Medicare?” A variable for *health insurance* (yes/no) was derived from the combined response data of 2011–2014.

Other sample characteristics included in the analysis were *Internet use* and *Internet access*. To determine *Internet use* (yes/no) participants were asked: “Do you ever go online to access the Internet or World Wide Web, or to send and receive e-mail?” *Internet access* (dial-up, broadband, cellular, or wireless networks) was assessed by a follow-up question: “When you use the Internet, do you access it through [dial-up, broadband, cellular, or wireless networks]?”

### Analytic methods

All analyses were conducted in STATA 14 using survey weighting and jackknife variance estimations provided by HINTS [15]. Analyses were first performed using the combined data from 2011 and 2012 to replicate the findings from Volkman et al. 2014 and ensure model fidelity. These analyses were then extended across 2011–2014, thereby including all currently available data.

Maintaining fidelity to the original paper [13], the data was analyzed describing the first source chosen by participants for health information (Table 1). Weighted descriptive statistics were analyzed for participants who reported a doctor/healthcare provider as their first source of health information (Table 2). Individual logistic regression models were created to explore the association between first source of health information and demographic characteristics as in *Volkman* et al. *2014*. A weighted multivariable logistic regression model was created to explore the adjusted association between first source of health information and demographic characteristics (Table 3). A category for missing data was included in the analysis of the descriptive statistics but was excluded from the logistic model.

**Table 1:** First Health Information Source

	Data Years 2011–2012 (*N* = 5307)			Data Years 2011–2014 (*N* = 10,024)		
Source	N	Weighted %	95% CI	N	Weighted %	95% CI
Internet	3315	67.63	65.89–69.33	6353	68.72	67.35–70.05
Doctor/healthcare provider	937	15.66	14.17–17.27	1798	15.26	14.21–16.37
Publications	652	9.39	8.38–10.52	1138	8.89	8.12–9.72
Family/friends/co-workers	235	4.95	4.17–5.87	444	4.97	4.32–5.72
Other	72	0.99	0.66–1.47	108	0.85	0.63–1.15
Telephone service	40	0.61	0.40–0.94	73	0.55	0.39–0.76
Cancer organization	17	0.26	0.14–0.49	30	0.24	0.14–0.43
Library	21	0.23	0.14–0.39	45	0.30	0.21–0.42
Complementary or alternative medicine practitioner	18	0.28	0.15–0.50	35	0.23	0.14–0.36

*CI* Confidence Interval

**Table 2:** Characteristics of Respondents Choosing Doctor/Healthcare Provider as First Source of Health Information

	Data Years 2011–2012 (*n* = 937)			Data Years 2011–2014 (*n* = 1789)		
	N	Weighted %	95% CI	N	Weighted %	95% CI
Gender						
Male	385	43.16	38.56–47.88	742	45.42	41.98–48.91
Female	531	53.81	48.90–58.64	1014	51.95	48.36–55.53
Missing	21	3.03	1.26–7.13	42	2.63	1.48–4.63
Age						
18–45	150	28.62	24.00–33.74	271	29.20	25.50–33.21
46–64	347	34.66	30.46–39.11	675	34.03	31.09–37.11
65–75	241	18.14	15.47–21.16	441	16.49	14.69–18.47
76–99	179	14.71	12.44–17.30	345	15.08	13.26–17.09
Missing	20	3.87	1.30–10.92	66	5.19	3.18–8.36
Rurality						
Yes	21	2.32	1.37–3.91	39	2.34	1.47–3.69
No	916	97.68	96.09–98.63	1759	97.66	96.31–98.53
Missing	–	–	–	–	–	–
Cancer history						
Yes	186	13.50	11.36–15.96	364	13.45	11.87–15.20
No	746	86.12	83.67–88.25	1413	84.98	83.01–86.75
Missing	5	0.39	0.14–1.05	21	1.58	0.78–3.15
General health						
Excellent	71	8.71	6.00–12.46	131	8.52	6.62–10.90
Very good	302	31.08	26.58–35.96	578	32.06	28.84–35.46
Good	347	34.63	30.25–39.27	655	36.73	33.29–40.32
Fair	141	16.47	12.83–20.90	279	14.78	12.29–17.66
Poor	52	6.48	3.58–11.45	98	5.04	3.34–7.53
Missing	24	2.64	1.60–4.33	57	2.88	2.02–4.08
Income						
Less than $20,000	234	26.40	21.31–32.21	466	25.56	22.13–29.32
$20,000 to $49,999	258	27.10	22.54–32.21	485	26.57	23.31–30.11
$50 k or above	311	34.14	29.91–38.65	577	34.25	31.15–37.49
Missing	134	12.35	9.31–16.21	270	13.63	11.46–16.13
Race/Ethnicity						
White, NH	532	58.93	53.77–63.90	912	56.04	52.30–59.71
Black, NH	143	9.18	7.13–11.73	290	10.64	8.58–13.14
Hispanic	111	16.36	12.42–21.25	245	15.31	12.56–18.53
Other, NH	63	6.74	4.44–10.10	111	5.63	4.12–7.63
Missing	88	8.80	6.48–11.83	240	12.39	10.14–15.05
Education						
Less than high school	118	23.11	17.99–29.16	239	20.42	17.14–24.15
High school graduate	240	23.01	19.23–27.27	431	21.77	19.07–24.73
Some college	292	31.83	27.26–36.79	570	31.00	27.57–34.65
College or above	266	20.39	17.46–23.66	494	23.23	20.61–26.08
Missing	21	1.67	0.97–2.86	64	3.57	2.52–5.04
Health insurance						
Yes	855	86.04	80.79–90.02	1638	87.10	83.88–89.76
No	65	10.74	7.54–15.08	122	9.76	7.53–12.57
Missing	17	3.22	1.10–9.11	38	3.13	1.68–5.77
Veteran status						
Veteran	139	11.45	9.04–14.39	264	11.52	9.68–13.67
Non-veteran	735	80.64	76.31–84.34	1391	80.30	77.24–83.03
Missing	63	7.91	5.27–11.72	143	8.18	6.24–10.64
Internet use						
Yes	550	61.81	56.45–66.90	1042	61.36	57.74–64.85
No	386	38.17	33.07–43.53	744	37.97	34.54–41.52
Missing	1	0.03	0.00–0.19	12	0.67	0.24–1.91
Access to Internet						
Telephone line	52	5.10	3.15–8.14	93	4.70	3.40–6.46
Broadband	359	37.47	32.29–42.96	645	38.52	34.84–42.34
Cellular network	166	22.29	17.49–27.95	370	25.37	21.81–29.29
Wireless network	274	31.30	26.59–36.43	565	35.61	31.90–39.49
Missing	12	5.12	1.58–15.31	27	3.77	1.62–8.50
Data Year						
2011	436	42.15	37.45–47.01	436	21.91	19.36–24.68
2012	501	57.85	52.99–62.55	501	30.06	26.64–33.72
2013	–	–	–	408	23.11	20.15–26.35
2014	–	–	–	453	24.92	21.87–28.25

*CI* Confidence Interval, *NH* Non-Hispanic

**Table 3:** Model of Seekers Choosing Doctor/Healthcare Provider First For 2011–2014

	Healthcare Provider N (weighted %)	Other Sources N (weighted %)	Unadjusted OR (95% CI), *p*-value	Adjusted OR (95% CI), *p*-value
Gender				
Male	742 (15.25)	3005 (84.75)	–	–
Female	1014 (14.98)	5080 (85.02)	0.98 (0.83–1.16), 0.803	0.85 (0.67–1.07), 0.156
Age				
18–45	271 (9.33)	2766 (90.67)	–	–
46–64	675 (15.28)	3471 (84.72)	1.75 (1.42–2.17), 0.000^c^	1.62 (1.26–2.07), 0.000 ^c^
65–75	441 (25.50)	1260 (74.50)	3.33 (2.65–4.17), 0.000^c^	2.15 (1.55–3.00), 0.000 ^c^
76–99	345 (38.87)	525 (61.13)	6.18 (4.81–7.94), 0.000^c^	2.94 (2.08–4.17), 0.000 ^c^
Rurality				
No	1759 (15.17)	8099 (84.83)	–	–
Yes	39 (20.18)	127 (79.82)	1.41 (0.79–2.53), 0.243	1.31 (0.65–2.64), 0.440
Cancer history				
No	1413 (14.23)	7104 (85.77)	–	–
Yes	364 (24.64)	1076 (75.36)	1.97 (1.66–2.34), 0.000^c^	1.19 (0.94–1.51), 0.149
General health				
Excellent	131 (10.74)	1006 (89.26)	–	–
Very good	578 (13.07)	3031 (86.93)	1.25 (0.90–1.73), 0.177	1.04 (0.71–1.52), 0.851
Good	655 (15.76)	2912 (84.24)	1.56 (1.13–2.14), 0.007^b^	0.95 (0.65–1.39), 0.794
Fair	279 (20.65)	932 (79.35)	2.16 (1.50–3.13), 0.000^c^	0.96 (0.59–1.54), 0.854
Poor	98 (35.98)	184 (64.02)	4.67 (2.55–8.55), 0.000^c^	2.22 (0.77–6.43), 0.141
Income				
Less than $20,000	466 (23.06)	1305 (76.94)	–	–
$20,000 to $49,999	485 (16.21)	2122 (83.79)	0.65 (0.50–0.84), 0.001^b^	0.87 (0.62–1.22), 0.427
$50 k or above	577 (10.68)	4019 (89.32)	0.40 (0.32–0.50), 0.000^c^	0.72 (0.51–1.02), 0.062
Race/Ethnicity				
White, NH	912 (13.02)	5112 (86.98)	–	–
Black, NH	290 (17.14)	1066 (82.86)	1.38 (1.03–1.85), 0.029^a^	1.46 (1.01–2.11), 0.043^a^
Hispanic	245 (19.97)	946 (80.03)	1.67 (1.28–2.18), 0.000^c^	1.26 (0.90–1.75), 0.178
Other, NH	111 (13.49)	519 (86.51)	1.04 (0.71–1.52), 0.833	1.24 (0.76–2.02), 0.380
Education				
Less than high school	239 (35.60)	409 (64.40)	–	–
High school graduate	431 (18.73)	1289 (81.27)	0.42 (0.30–0.57), 0.000^c^	0.68 (0.42–1.11), 0.123
Some college	570 (13.85)	2465 (86.15)	0.29 (0.22–0.39), 0.000^c^	0.67 (0.41–1.10), 0.115
College or above	494 (9.48)	3895 (90.52)	0.19 (0.14–0.25), 0.000^c^	0.50 (0.29–0.865, 0.011^a^
Health insurance				
No	122 (10.39)	945 (89.61)	–	–
Yes	1638 (15.76)	7179 (84.24)	1.61 (1.17–2.21), 0.003^b^	1.97 (1.28–3.04), 0.002^b^
Veteran status				
Non-veteran	1391 (14.03)	7079 (85.97)	–	–
Veteran	264 (19.05)	866 (80.95)	1.44 (1.15–1.82), 0.002 ^b^	1.05 (0.77–1.44), 0.742
Internet use				
No	744 (43.71)	974 (56.29)	–	–
Yes	1042 (10.83)	7214 (89.17)	0.16 (0.13–0.19), 0.000^c^	0.34 (0.25–0.46), 0.000^c^
Data Year				
2011	436 (13.62)	2263 (86.38)	–	–
2012	501 (17.58)	2107 (82.42)	1.35 (1.08–1.70), 0.010^a^	1.22 (0.92–1.62), 0.157
2013	408 (14.84)	1738 (85.16)	1.11 (0.88–1.38), 0.383	0.95 (0.69–1.31), 0.737
2014	453 (14.86)	2118 (85.14)	1.11 (0.89–1.38), 0.368	1.14 (0.86–1.52), 0.358

^a^= significant at the 0.05 level; ^b^ = significant at the 0.01 level, ^c^ = significant at the 0.001 level

*OR* Odds Ratio, *CI* Confidence Interval, *NH* Non-Hispanic

## Results

Our analysis describing the first source of health information showed that the majority of survey respondents continue to report using the Internet as a first source of health information (*n* = 6353, 68.72%) followed by doctor/healthcare provider (*n* = 1798, 15.26), publications (*n* = 1138, 8.89%), and family/friends/co-workers (*n* = 444, 4.97%).

The respondents who more frequently selected doctor/healthcare provider as a first source the most recent time they looked for health information were female (*n* = 1014, 51.95%), ages 46–64 (*n* = 675, 34.03%), non-rural (*n* = 1795, 97.66%), had an income of $50,000 or above (*n* = 577, 34.25%), non-Hispanic white (*n* = 912, 56.04%), had some college education (*n* = 570, 31.00%), and were non-veterans (*n* = 1391, 80.30%). These respondents also had no history of cancer (*n* = 1413, 84.98%), reported very good (*n* = 578, 32.06) or good health (*n* = 655, 36.73), and had health insurance (*n* = 1638, 87.10%). These respondents used the Internet (*n* = 1042, 61.36%) and accessed the internet through broadband (*n* = 645, 38.52%) or wireless networks (*n* = 565, 35.61%). (n = 1042, 61.36%).

An adjusted logistic regression showed significant differences in the likelihood of a respondent reporting doctors as first sources of health information on the basis of age, race, education, health insurance, and Internet use. Individuals who chose doctor/healthcare provider as a first source were more likely to be aged 46–64 [OR = 1.62, *p* = 0.000], 65–75 [OR = 2.15, p = 0.000], or 76–99 [OR = 2.94, p = 0.000] than to be in the 18–45 year age group. Those who chose a doctor as the first source of health information were more likely to be non-Hispanic black [OR = 1.46, *p* = 0.043], more likely to have health insurance [OR = 1.97, *p* = 0.002], less likely to have college education or more [OR = 0.50, *p* = 0.011], and less likely to use the Internet [OR = 0.34, p = 0.000]. Significant differences in the unadjusted logistic regression models for cancer history, general health, income, race (Hispanic), education (high school and some college), veteran status, and data year were attenuated when the logistic regression model was adjusted, similar to the prior analysis.

## Discussion

Adults of all age groups older than 18–45 had higher odds of listing their doctor as their first source of health information, and these odds increased with successive age groups. This finding contrasts with prior research in which the OR for the age group 46–64 was not significant [13]. Trust may play a role, as prior research revealed that older adults are less likely to trust information from the Internet, television, magazines, and newspapers than young adults, while trusting doctors the most of any source [18]. Young adults may be less likely to go to physicians as a source of health information as this age group is historically less likely to use primary care services or have insurance coverage [19], although the uninsured rate has decreased since open enrollment for the Affordable Care Act began [20]. Young adults are also more likely to be Internet users, and use the Internet as an initial source of health information [21].

In our updated analyses but not in the original research [13], respondents who were black had higher odds of noting that a doctor was their first source of health information. This finding is in contrast with the results of prior research reporting that black patients do not trust doctors as much as white patients [22]. Since the Affordable Care Act was implemented, there has been a significant reduction in racial disparities in health care access, utilization, and rates of being uninsured [23]. This reduction in access disparities may have played a role in the increase in black patients choosing doctors as a first source of medical information, as they were more readily able to see doctors. The attitudes of black patients toward doctors may be improved by this increased access to health care and communication with doctors [24].

Our results also show that respondents with more education had lower odds of using their doctor as a first source of health information, even though prior research demonstrates that college-educated adults are more likely to have a usual place of health care [25]. Prior research has shown that those with higher levels of education were more trusting of the Internet, magazines, and newspapers as sources of health information than those with lower levels [18]. These respondents may choose the internet first, similar to research corroborating that those with higher education levels more frequently seek health information on the Internet [26]. In our analyses, those who use the Internet have lower odds of seeking a doctor as the first source of health information. Using the Internet for information may lead to self-diagnosis, encouraging misdiagnosis and inappropriate medical advice [27]. HINTS does not ask questions regarding the specific internet sources used and trusted; future research could elaborate on the legitimacy of trusted internet health information sources.

In the original analysis [13], people who rated their general health as good had lower odds of choosing a doctor and those who rated their health as poor had higher odds of choosing a doctor as their first health information source than those with excellent health, however, the impact of health status on choice of health information source was no longer significant with the addition of 2 years of data. This could be due to an increase in those with poor health using the internet or other sources or people with good health seeking information from doctors more. Healthy people may be seeking information from doctors first more than in previous years due to increased access to care as the uninsured rate has decreased [20]. Other prior research provides evidence that people with poor health are more likely to seek health information on the Internet than those with good health, and the internet may be displacing doctors as a primary source in this group [28]. People with poor health may have chronic conditions that require more health information or more frequent information than regular patients, and may use multiple information sources [29]. Doctors can assist these patients by helping verify information and guiding them on where to find credible sources [30].

This analysis was strengthened by the addition of an extra 2 years of data, doubling the sample size and allowing for analysis of changes in significance. However, a limitation that persists is the cross-sectional nature of the data because within-person trends over time cannot be assessed. A second limitation is the fact that these are self-reported data, as people may not accurately recall whether they used certain information sources. HINTS only surveys U.S. households, so these findings may not be generalizable to populations outside the United States; there may be differences in trust and use of internet health sources in other countries. Further, the specific publications or Internet sources of health information were not assessed, so the credibility of the sources remains unknown. Finally, we did not know whether the doctors mentioned were primary care or specialty doctors, and this information may add depth to the analysis. Future research could elaborate on both the use and trust of more specific sources of information.

## Conclusion

Doctors are the most trusted sources of health information yet the results of a recent survey of health information sources show that 69% of adults in the U.S. reportedly chose the Internet and 15% chose healthcare providers as their first source of health information when they had a medical problem. Those more likely to choose doctors as an initial source of health information were older than age 45, more likely to have health insurance, more likely to be black, less likely to be college educated, and less likely to use the Internet. Doctors may need to provide more information to these patients than those who use the Internet, however, they may need to spend more time verifying the accuracy of information provided to those who choose the Internet first.

## Abbreviations

HINTS: Health Information National Trends Survey
HMO: Health maintenance organization
NCI: National cancer institute
OR: Odds ratio
US: United States
VA: Veteran’s affairs
